# Editorial: Elevation Gradients: Microbial Indicators of Climate Change?

**DOI:** 10.3389/fmicb.2019.02405

**Published:** 2019-10-18

**Authors:** Rosa Margesin, Maria Alicja Niklinska

**Affiliations:** ^1^Institute of Microbiology, University of Innsbruck, Innsbruck, Austria; ^2^Institute of Environmental Sciences, Jagiellonian University, Kraków, Poland

**Keywords:** soil, rhizosphere, microbial communities, elevation, climate change

Climate change is one of the largest challenges facing our planet. The Fifth Assessment Report of the Intergovernmental Panel on Climate Change estimated that by the end of the twenty-first century, under RCP8.5 scenario, the global average surface temperature will increase in the range of 2.6–4.8°C. This climate change will produce an immediate rising of soil temperature, which, along with other alterations, such as drought and precipitation pattern, increasing carbon dioxide levels or nitrogen deposition, will affect the structure, diversity and abundance of soil microbial communities.

Elevation gradients have been regarded as especially interesting to study the effects of global warming on terrestrial ecosystems. Such gradients represent powerful “natural experiments” to gain information on the response of soil microbial communities to variations in temperature and the associated climatic factors. Elevation gradients are characterized by strong changes in climate and biotic characteristics over short distances and are thus useful to better predict and help mitigate the effects of climate change. In addition, the altitudinally-defined vegetation belts on mountain slopes are counterparts to the latitudinally-controlled climatic zones. In the last years, a number of studies have demonstrated changes in soil microbial diversity, community structure, abundance, and several activities (e.g., enzymes, soil organic matter decomposition, respiration) along elevation gradients, which were significantly correlated with environmental parameters, including climate.

The present Research Topic on “Elevation Gradients: Microbial Indicators of Climate Change?” comprises 4 original articles on the response of soil microorganisms to climate change, contributed by 42 authors.

Adamczyk et al. provide the first insights into the molecular diversity and community structure of the unexplored soil microbiome of 10 mountain summits in the European (Swiss) Alps. These summits belong to the three Swiss target regions of the worldwide long-term monitoring network “Global Observation Research Initiative in Alpine environments (GLORIA),” which was initiated to gain knowledge on the impact of climate change on mountain ecosystems. The 10 summits range from the lower alpine to the nival zone and span an elevation gradient of 850 m [2,360–3,212 m above sea level (a.s.l.)]. The study demonstrated highly diverse microbial communities in the summit soils as a result of highly variable local environmental conditions, such as soil properties, parent material, climatic variables, and vegetation. The strong influence of environmental changes on bacterial communities indicates that they will be strongly affected by future climate change (associated temperature increase and upwards migration of vegetation).

The fungal class Archaeorhizomycetes is widespread in soil environments, associated with plant roots and rhizosphere, and has putative saprotrophic activity. However, the absence of systematic sampling of fungal communities along environmental gradients makes the ecological role and the trophic status of Archaeorhizomycetes in terrestrial ecosystems still elusive. Pinto-Figueroa et al. examined the environmental requirements and the biotic relationships with other soil fungi and plants. The metagenomic DNA data from the soils of 103 sites in the Swiss Alps along an elevation gradient spanning >2,500 m (374–3,210 m a.s.l.) demonstrated that the variation in the fungal abundance was explained by a range of climatic and edaphic factors. Co-abundance patterns between Archaeorhizomycetes and other saprotrophic and ectomycorrhizal taxa were recognized. The potential role of Archaeorhizomycetes in biological rock weathering, because of high correlation to soil redox parameters, is also discussed.

Microbial communities in bulk and rhizosphere soil of *Ranunculus glacialis* along a high Alpine altitudinal gradient (2,600–3,400 m a.s.l.) in the Central Alps in Austria were studied in detail by Praeg et al. No overall altitudinal pattern of microbial community structure and diversity was detected. The biggest differences in the abundance of specific biomarker taxa were observed for the mid-altitudinal transition zone (3,000–3,100 m a.s.l.) which corresponds with the summer snow line. Prokaryotes were mainly influenced by soil temperature and pH. Fungal communities were also related to altitude and showed often unique occurrence at the highest elevations in the nival zone. The rhizosphere of *R. glacialis* significantly influenced the community composition of prokaryotes but not of fungi.

The effects of snowpack decline and plant root on soil microbial communities and nitrogen mineralization were studied *in situ* for 29 months across an elevation gradient in northern hardwood forest, in the White Mountain National Forest in New Hampshire, USA. Sorensen et al. showed that the differences in winter snow depth and soil frost explained variation in bacterial community composition along elevation, whereas variation in fungal community composition was best explained by standing root biomass. The potential soil N mineralization rates were related to saprotrophic and ectomycorrhizal abundance, while soil nitrification rates were related to the abundance of ammonia-oxidizing bacteria and archaea. The results suggest that a declining winter snowpack and its effect on plant roots each impact the diversity and abundance of soil bacteria and fungi that can interact to determine rates of soil N cycling in northern forest ecosystems.

In summary, the articles included in this Research Topic demonstrate the importance of elevation gradients for the estimation of the effects of climate change on soil microbial parameters and thus soil processes. The results of such studies allow to predict that climate change will have direct (temperature, water content) and indirect (soil parameters, plant species, and their rhizosphere) impacts on soil microbial community structure and diversity and, as a consequence, could change soil processes, especially cycling of carbon and nitrogen, which in turn will significantly enhance the effects of climate change.

## Author Contributions

RM and MN wrote the manuscript. All authors read and approved the final manuscript.

### Conflict of Interest

The authors declare that the research was conducted in the absence of any commercial or financial relationships that could be construed as a potential conflict of interest.

